# Delineating the psychiatric and behavioral phenotype of recurrent 2q13 deletions and duplications

**DOI:** 10.1002/ajmg.b.32627

**Published:** 2018-03-31

**Authors:** Kate Wolfe, Andrew McQuillin, Viola Alesi, Elise Boudry Labis, Peter Cutajar, Bruno Dallapiccola, Maria Lisa Dentici, Anne Dieux‐Coeslier, Benedicte Duban‐Bedu, Tina Duelund Hjortshøj, Himanshu Goel, Sara Loddo, Deborah Morrogh, Anne‐Laure Mosca‐Boidron, Antonio Novelli, Laurence Olivier‐Faivre, Jennifer Parker, Michael J. Parker, Christine Patch, Anna L. Pelling, Thomas Smol, Zeynep Tümer, Olivier Vanakker, Arie van Haeringen, Clémence Vanlerberghe, Andre Strydom, David Skuse, Nick Bass

**Affiliations:** ^1^ Molecular Psychiatry Laboratory, Division of Psychiatry University College London London United Kingdom; ^2^ Medical Genetics Unit, Medical Genetics Laboratory Bambino Gesù Pediatric Hospital, IRCCS Rome Italy; ^3^ Institut de génétique médicale, CHU Lille Lille France; ^4^ Nottinghamshire Healthcare NHS Foundation Trust Nottingham United Kingdom; ^5^ Service de génétique clinique, CHU Lille Lille France; ^6^ EA7364, RADEME, Université de Lille Lille France; ^7^ Centre de génétique chromosomique, Hopital Saint‐Vincent de Paul Lille France; ^8^ Kennedy Center, Department of Clinical Genetics Copenhagen University Hospital, Rigshospitalet Copenhagen Denmark; ^9^ Hunter Genetics Waratah New South Wales Australia; ^10^ University of Newcastle Callaghan New South Wales Australia; ^11^ North East Thames Regional Genetics Service Laboratory London United Kingdom; ^12^ Service de Cytogénétique, Plateau technique de Biologie CHU Dijon France; ^13^ Centre de référence Anomalies du développement et Syndromes malformatifs, FHU TRANSLAD CHU Dijon France; ^14^ Sheffield Clinical Genetics Service, Sheffield Children's Hospital, Western Bank Sheffield United Kingdom; ^15^ King's College London, Florence Nightingale Faculty of Nursing and Midwifery London United Kingdom; ^16^ Genomics England, Dawson Hall, Charterhouse Square London United Kingdom; ^17^ Information Officer, Unique – The Rare Chromosome Disorder Support Group (www.rarechromo.org), The Stables, Station Road West Oxted, Surrey United Kingdom; ^18^ Center for Medical Genetics Ghent University Hospital Ghent Belgium; ^19^ Department of Clinical Genetics Leiden University Medical Center Leiden The Netherlands; ^20^ Department of Forensic and Neurodevelopmental Science Institute of Psychiatry, Psychology and Neuroscience, Kings College London London United Kingdom; ^21^ Behavioural and Brain Sciences Unit Institute of Child Health, University College London London United Kingdom

**Keywords:** attention deficit hyperactivity disorder, autism spectrum disorders, copy number variants, developmental delay, intellectual disabilities

## Abstract

Recurrent deletions and duplications at the 2q13 locus have been associated with developmental delay (DD) and dysmorphisms. We aimed to undertake detailed clinical characterization of individuals with 2q13 copy number variations (CNVs), with a focus on behavioral and psychiatric phenotypes. Participants were recruited via the Unique chromosomal disorder support group, U.K. National Health Service Regional Genetics Centres, and the DatabasE of genomiC varIation and Phenotype in Humans using Ensembl Resources (DECIPHER) database. A review of published 2q13 patient case reports was undertaken to enable combined phenotypic analysis. We present a new case series of 2q13 CNV carriers (21 deletion, 4 duplication) and the largest ever combined analysis with data from published studies, making a total of 54 deletion and 23 duplication carriers. DD/intellectual disabilities was identified in the majority of carriers (79% deletion, 70% duplication), although in the new cases 52% had an IQ in the borderline or normal range. Despite the median age of the new cases being only 9 years, 64% had a clinical psychiatric diagnosis. Combined analysis found attention deficit hyperactivity disorder (ADHD) to be the most frequent diagnosis (48% deletion, 60% duplication), followed by autism spectrum disorders (33% deletion, 17% duplication). Aggressive (33%) and self‐injurious behaviors (33%) were also identified in the new cases. CNVs at 2q13 are typically associated with DD with mildly impaired intelligence, and a high rate of childhood psychiatric diagnoses—particularly ADHD. We have further characterized the clinical phenotype related to imbalances of the 2q13 region and identified it as a region of interest for the neurobiological investigation of ADHD.

## 1 INTRODUCTION

Chromosomal microarray analysis technologies have led to the discovery of chromosomal imbalances across the human genome. These imbalances, or copy number variations (CNVs), comprise deletions or duplications affecting single or multiple genes on sections of the chromosome. CNVs occur as part of natural variation and can be benign in nature. Typically, it is the rare CNVs, occurring in less than 1% of the population, that are enriched in individuals with neurodevelopmental and neuropsychiatric phenotypes (Iyer & Girirajan, 2015). Several challenges, however, remain in CNV interpretation. CNVs frequently display reduced penetrance, meaning not everyone with the CNV displays the disease phenotype, and/or variable expressivity, whereby the severity of the phenotype differs between CNV carriers (Rosenfeld, Coe, Eichler, Cuckle, & Shaffer, 2013).

Certain regions of the genome are more liable to recurrent CNVs. These regions are flanked by segmental duplications (SDs), also known as low copy repeats, which are prone to mismatching of repetitive DNA sequences mediated by non‐allelic homologous recombination (Malhotra & Sebat, 2012). Investigation of chromosomal rearrangements in regions of SD, identified 2q13 CNVs in patients with developmental disorders (Rudd et al., 2009). However, the pathogenicity of these CNVs was initially described as of uncertain significance. This was due to a 2q13 duplication also being found in a healthy control (Rudd et al., 2009), and the findings from a previous study that the same 2q13 deletion in two siblings with developmental problems had been inherited from an unaffected parent (Bisgaard et al., 2007).

Analysis of larger samples revealed that 2q13 CNVs are associated with an increased risk of developmental delay (DD) and intellectual disabilities (IDs). Cooper et al. (2011) reported 12 deletions and 9 duplications in cases with developmental disorders (*N* = 15,767) and 1 deletion and 0 duplications in controls (*N* = 8,329). They found an enrichment of the deletion (*p* = .032) and duplication (*p* = .022) in cases, as compared to controls. The deletion was associated with cardiovascular disorders, whereas the duplication was associated with craniofacial features (Cooper et al., 2011). Yu et al. (2012) described the phenotype of five 2q13 patients alongside 14 additional cases from a literature review, concluding that 93% of individuals had impaired development and 63% had facial dysmorphisms. Some of these patients had a diagnosis of autism spectrum disorder (ASD) or attention deficit hyperactivity disorder (ADHD), although many were too young for clinical assessment, or it was unclear whether assessments had taken place.

Costain et al. (2013) found 2q13 CNVs to be significantly associated with schizophrenia (*p* = .0002) in a community‐based schizophrenia cohort (*N* = 459), as compared to a large population‐based control sample (*N* = 23,838). They identified three 2q13 CNV carriers (one deletion, two duplications) in cases and four CNV carriers (one deletion, three duplications) in controls (Costain et al., 2013). However, subsequent case‐control studies in larger schizophrenia patient cohorts have failed to find a significant association at the 2q13 locus (Marshall et al., 2017; Rees et al., 2016). In a follow up study Costain et al. (2014) undertook detailed phenotyping with two unrelated 2q13 duplication carriers and their families, identified in the 2013 study. Four family members, from one patient pedigree, also carried the duplication and this co‐segregated with a neuropsychiatric phenotype. There was a variable psychiatric phenotype, with one psychotic disorder, two major mood and/or anxiety disorders, and one mood and/or anxiety disorder and obsessive compulsive disorder (OCD). The original patient with schizophrenia also had OCD. None of these individuals had significant DD, ASD or facial dysmorphisms, although three of the family members and one patient had learning difficulties (Costain et al., 2014).

Riley et al. (2015) identified three 2q13 deletion carriers and one 2q13 duplication carrier, and compared the phenotype with previous published cases. They concluded that congenital heart defects, hypotonia, dysmorphic features, and abnormal head size are common in deletion carriers and DD, dysmorphic features, and abnormal head size are common in duplication carriers. No ASD or psychiatric phenotype was described in these patients, likely because they were too young for clinical assessment (Riley et al., 2015). Finally, Hladilkova et al. (2015) described two additional 2q13 deletion patients, one of whom had ASD and ADHD.

A large study of rare CNVs estimated the rate of occurrence of 2q13 deletions and duplications in healthy controls (0.004% deletions, 0.015% duplications), schizophrenia patients (0.015% deletions, 0.02% duplications), and a mixed developmental disorders (predominantly DD/ID and ASD) cohort (0.057% deletions, 0.022%, duplications) (Rees et al., 2016). This suggests that 2q13 CNVs can be observed in healthy controls, but are more common in schizophrenia cohorts, and even more common in patients with developmental disorders. The 2q13 CNV is now understood to be a susceptibility locus, which describes a CNV that can be inherited from a healthy or mildly affected parent, but is enriched in individuals with various developmental disorders (De Wolf, Brison, Devriendt, & Peeters, 2013).

A limitation of current 2q13 CNV literature is that few studies have undertaken comprehensive behavioral and psychiatric phenotyping, so the full extent of the neuropsychiatric risk associated with these CNVs remains unclear. We aimed to further delineate the 2q13 CNV profile by undertaking deep phenotyping comprising: developmental, medical, dysmorphic, behavioral, and psychiatric features.

## 2 MATERIALS AND METHODS

### 2.1 Participant recruitment

In order to maximize recruitment of patients with this rare CNV a multi‐faceted approach to recruitment was employed. Unique is a U.K.‐based support group (http://www.rarechromo.org), working internationally to inform and support anyone affected by a rare chromosome or single gene disorder and with professionals involved in their care. The Unique Information Officer identified and emailed registered contacts of Unique members with 2q13 CNVs. Information was provided about the study, and contacts were encouraged to contact the study team if they wanted to participate. Patients with 2q13 deletions were also identified via two National Health Service (NHS) Regional Genetics Centres (RGCs)—the North East and South East Thames RGCs. Clinicians were approached in the first instance and where appropriate invitations to participate in the study were sent to the patient contact via letter.

Additionally, patients with 2q13 CNVs on the DatabasE of genomiC varIation and Phenotype in Humans using Ensembl Resources (DECIPHER) database (https://decipher.sanger.ac.uk/) were identified and further phenotypic information was sought from responsible clinicians. One participant was also included from a previous investigation of CNVs in adults recruited from ID psychiatry services (Wolfe et al., 2017). Informants or clinicians were asked whether the participant had taken place in previous research projects, and to the best of our knowledge none of the other patients have been presented in previous studies. Ethical approval for the study was attained from the North Wales Research Ethics Committee West, reference 11/WA/0370.

### 2.2 Phenotyping and analysis protocol

All participants recruited through Unique and the NHS RGCs (*n* = 10), referred to as the detailed phenotyping group, underwent deep phenotyping. Clinical data, including medical and psychiatric history, was collected from a parent in a face‐to‐face interview. This was conducted by KW and interviews were undertaken in person for U.K. recruits and via Skype for overseas recruits. Responsible DECIPHER contacts were contacted via email to provide further phenotypic data about their patients and anonymized data was collected via the University College London (UCL) web‐based survey tool Opinio (*n* = 15).

All phenotypes were converted to human phenotype ontology (HPO) terms for presentation in the manuscript (Köhler et al., 2017). The level of ID was taken from available medical records or reported by informants or clinicians and was categorized in accordance with the HPO criteria: borderline intellectual disability (IQ 70–79), mild intellectual disability (IQ 50–69), moderate intellectual disability (IQ 35–49), and severe intellectual disability (IQ 20–34).

Clinical psychiatric diagnoses were taken from available medical records, or from informant or clinician reports. Additional psychiatric and behavioral phenotyping was undertaken for the detailed phenotyping group. Psychiatric phenotyping was undertaken using the Mini Psychiatric Assessment Schedule for Adults with Developmental Disabilities (Mini PAS‐ADD) for participants over 18 years of age, and the Child and Adolescent Psychiatric Assessment Schedule (ChA‐PAS) for those under 18. These assessments provide threshold scores for psychiatric symptoms that are likely to warrant a diagnosis in conjunction with a clinical psychiatric assessment (Moss et al., 1998). The Mini PAS‐ADD includes ASD screening, but does not include an ADHD assessment. The ADHD section of the CHA‐PAS requires a second informant, who is familiar with the individual in other contexts (typically a teacher). It was not possible to interview a second informant for the ChA‐PAS, so both sections were completed by the primary informant.

Behavioral phenotyping was undertaken using the behavior problems inventory—short form (BPI‐S). The BPI‐S provides frequency scores of self‐injurious and aggressive/destructive behaviors (Rojahn et al., 2012). Behaviors were reported as present if they occurred at a minimum of a weekly frequency on the BPI‐S measure, or were documented in the medical history. General observations for dysmorphic features were also made and photographs taken where consent was given. Dysmorphic features were independently verified by a second investigator (NB, Consultant Psychiatrist).

Data analysis and visualization was undertaken using R version 3.4.2 and the ggplot2, Rcmdr, and ontologyX packages (Fox, 2005; Greene, Richardson, & Turro, 2017; R Core Team., 2017; Wickham, 2009). For the breakdown of CNV carriers for each phenotype, deletion and duplication will be abbreviated to del and dup. Finally, a literature search was undertaken on PubMed using the search terms “2q13 deletion” and “2q13 duplication” to identify previously published 2q13 case reports for combined phenotypic analysis. The supplementary table detailing the phenotype of patients from previously published studies has been adapted from the table initially presented by Hladilkova et al. (permission via personal correspondence) (Hladilkova et al., 2015).

## 3 RESULTS

### 3.1 Sample description

**Figure 1:**
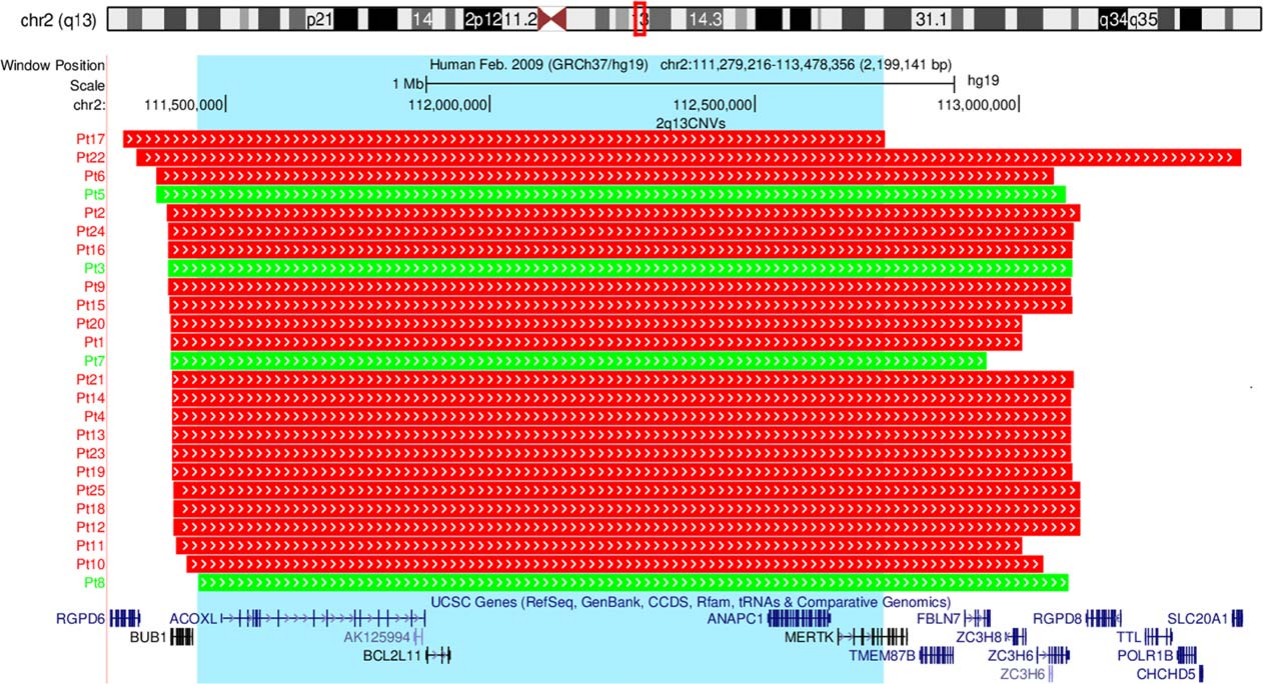
Chromosomal location of the CNV breakpoints for 2q13 CNV carriers (*n* = 25). The top of the image shows the location of the CNV region (highlighted by a red box) on a schematic of chromosome 2. The chromosomal breakpoints for each participant are shown by the red (representing CNV deletions) and green (representing CNV duplications) boxes. UCSC genes contained in the 2q13 region are shown. The blue highlighted box displays the 1.3 Mb region of overlap between CNVs. The image was exported from UCSC in chromosomal build GRCh37/hg19 [Color figure can be viewed at http://wileyonlinelibrary.com]

A total of 25 participants were recruited to the study, 10 from the Unique and NHS RGCs group and 15 from the Decipher group (64% male). The participants were predominantly children (92% <18 years of age, median age 9 years, range 4–42 years). The data set comprises 21 deletion and 4 duplication carriers. The CNVs ranged in size from 1.4 Mb to 2.1 Mb, with a 1.3 Mb region of overlap between all CNVs (chr2:111449141–112746937, hg19), see Figure 1. One family with an inherited 2q13 deletion is included in the case series, a father and two children, as removing the family did not change the results they are presented together with the rest of the cohort. Detailed phenotypic data for all cases is presented in Supporting Information Table S1.

### 3.2 Inheritance status

For 36% of participants the inheritance status was unknown (8 del, 1 dup). A further 20% (5 del) had de novo CNVs, 28% (5 del, 2 dup) had a paternally inherited CNV, 8% (1 del, 1 dup) had a maternally inherited CNV, and finally 8% (2 del) had inherited CNVs but the parental origin was unknown. Focusing on the 11 individuals with inherited 2q13 CNVs, four (33%, 2 del, 2 dup) had no family history of ID or mental health problems, four had a family history of ID and/or mental health problems (36%, 4 del), and three (25%, 2 del, 1 dup) had a family history of ID and/or mental health problems only on the side of the family from which the 2q13 CNV was not transmitted.

### 3.3 Intellectual and learning difficulties

Overall 76% of participants had DD (15 del, 4 dup). Just over half the participants had an IQ in the borderline or average range (52%, 10 del, 3 dup), and (32%, 8 del) had mild ID. There were no individuals with moderate ID and 12% had severe ID (2 del, 1 dup). We also asked informants or clinicians whether the participants had any other specific learning difficulties, four participants (16%) had dyslexia, two participants (8%) had dyscalculia, and two participants (8%) had an auditory processing disorder (all these were identified in del carriers only).

### 3.4 Psychiatric disorders and challenging behaviors

**Figure 2:**
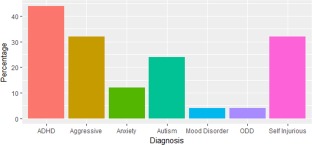
Clinically diagnosed psychiatric disorders and behavioral phenotypes in 2q13 deletion (*n* = 21) and duplication (*n* = 4) carriers. Y axis: percentage of participants with the diagnosis or behavior; X axis: ADHD—attention deficit hyperactivity disorder, Aggressive—aggressive behaviors, Anxiety—anxiety disorder, Autism—autism spectrum disorder, ODD—oppositional defiant disorder, Self‐injurious—self‐injurious behaviors [Color figure can be viewed at http://wileyonlinelibrary.com]

In total 64% of participants had a formal psychiatric diagnosis, among these 44% (9 del, 2 dup) had one diagnosis and 20% (4 del, 1 dup) had two. The most frequently diagnosed psychiatric disorder was ADHD (44%, 9 del, 2 dup), followed by ASD (24%, 5 del, 1 dup) and anxiety disorders (12%, 2 del, 1 dup). Both aggressive and self‐injurious behaviors were also identified in the participants, eight had aggressive behaviors (32%, all del) and eight had self‐injurious behaviors (32%, 7 del, 1 dup), for an overview see Figure 2.

Of the detailed phenotyping group (*n* = 10), five had no clinical psychiatric diagnosis. For two of these participants, both aged 6, ADHD was suspected, but the families were awaiting formal clinical assessment. Additionally, ASD was suspected for one of these participants. Taking into account the PAS‐ADD and ChA‐PAS thresholds, 9/10 individuals reached one or more of the thresholds. The most frequent thresholds met were anxiety disorder (60%, all del) and manic episode (60%, 5 del, 1 dup), followed by 20% each for ADHD, depressive disorder and psychosis (all del).

### 3.5 Medical and dysmorphology phenotypes

**Figure 3:**
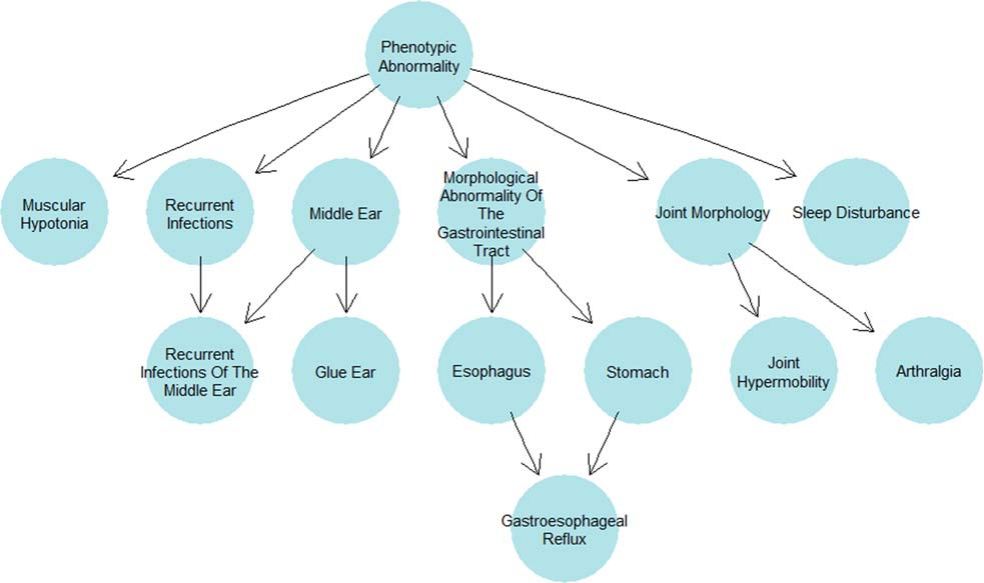
Human phenotype ontology tree plot with ancestral ontologies for the medical phenotypes occurring in more than three participants [Color figure can be viewed at http://wileyonlinelibrary.com]

**Figure 4:**
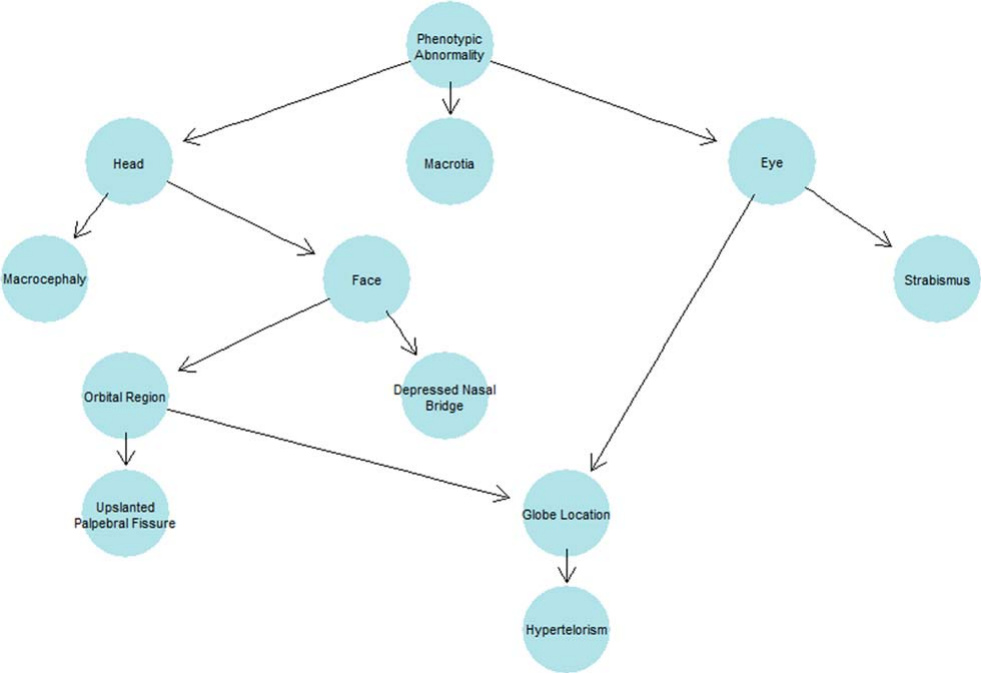
Human phenotype ontology tree plot with ancestral ontologies for the dysmorphology phenotypes occurring in more than three participants [Color figure can be viewed at http://wileyonlinelibrary.com]

The most commonly observed medical phenotypes were: glue ear (40%, 9 del, 1 dup), followed by muscular hypotonia (28%, 6 del, 1 dup), sleep disturbances (28%, 6 del, 1 dup), arthralgia (24%, 6 del), recurrent infections of the middle ear (20%, 4 del, 1 dup), joint hypermobility (20%, 5 del), and gastroesophageal reflux (16%, 4 del). The most commonly observed dysmorphology phenotypes were: macrotia (24%, 6 del), abnormality of the skull (24%, 3 del, 3 dup), macrocephaly (16%, 4 del), upslanted palpebral fissure (16%, 3 del, 1 dup), hypertelorism (16%, 4 del), strabismus (16%, 2 del, 2 dup), and depressed nasal bridge (16%, 4 del). See Figures 3 and 4 for an overview of the systems affected.

### 3.6 Combined phenotypic analysis

A review of the previous literature identified 11 published studies with phenotypic data for 2q13 deletion carriers (Bisgaard et al., 2007; Cooper et al., 2011; Costain et al., 2013; Hladilkova et al., 2015; Riley et al., 2015; Roberts, Hovanes, Dasouki, Manzardo, & Butler, 2014; Rudd et al., 2009; Russell et al., 2014; Vuillaume et al., 2014; Yu et al., 2016, 2012) and seven published studies with phenotypic data for 2q13 duplication carriers (Cooper et al., 2011; Costain et al., 2014, 2013; Riley et al., 2015; Roberts et al., 2014; Rudd et al., 2009; Yu et al., 2012) (some studies include both deletion and duplication carriers). Comprehensive phenotypic data for controls or unaffected parents was not available and is not considered in this analysis. Table 1 presents an overview of all known 2q13 deletion and duplication patient cases to date, including the participants from this study, and the phenotypes observed. Detailed phenotypic data for the patients described in these studies is available in Supporting Information Table S2.

**Table 1:** Summary of phenotypic observations in 2q13 deletion and duplication carriers (comprising the results of this study and available phenotypes from previously published studies)

	2q13 deletions	2q13 duplications
DD/ID	31/39 (79%)	14/20 (70%)
ASDs	9/27 (33%)	2/12 (17%)
ADHD	12/25 (48%)	3/5 (60%)
Dysmorphic features	33/41 (80%)	9/10 (90%)
Heart defect	11/35 (31%)	0/5 (0%)
Hypotonia	15/34 (44%)	3/7 (43%)
Seizures	8/31 (26%)	0/10 (0%)
Macrocephaly	10/35 (29%)	1/7 (14%)
Microcephaly	8/35 (23%)	2/7 (29%)

For each phenotype the number of patients with the diagnosis is displayed alongside the number of patients for which the phenotype was assessed. Thus, the denominator differs due to the varying availability of phenotypic information in published case studies. Percentages are provided for each individual phenotype. ADHD = attention deficit hyperactivity disorder; ASDs = autism spectrum disorders; DD = developmental delay; ID = intellectual disabilities

## 4 DISCUSSION

CNVs at the 2q13 locus are rare in the population, can be observed in healthy controls and transmitted from unaffected parents. Despite this, multiple studies have now shown that CNVs at 2q13 are risk factors for DD and dysmorphisms. This study represents the largest ever case series of 2q13 patients, comprising detailed phenotypic data for 25 new cases and combined analysis in 77 individuals, refining our understanding of the phenotypic associations of CNVs at the 2q13 locus.

DD was identified in 76% of participants in this study. Combined with data from existing studies 79% of deletion carriers and 70% of duplication carriers have DD/ID. We have further delineated this phenotype by investigating the level of ID, finding that the intellectual impairment is generally mild and 52% of participants have IQ in the borderline or average range. Only 12% of participants had severe ID, and data available for two of these participants revealed that both were referred for further exome sequencing investigations, due to the 2q13 CNV not being thought to fully explain their phenotype.

Combined analysis reveals that 80% of 2q13 deletion carriers and 90% of 2q13 duplication carriers have dysmorphic features. Deep phenotyping in the new cases showed that macrotia, abnormalities of the skull, macrocephaly, upslanted palpebral fissures, hypertelorism, strabismus, and depressed nasal bridge were common in deletion carriers. In duplication carriers, abnormalities of the skull and strabismus were observed. No other features achieved more than a single occurrence, however there were only four individuals who had 2q13 duplications. Combined analysis identified 29% of deletion carriers and 14% of duplication carriers as having macrocephaly and 23% of deletion carriers and 29% of duplication carriers as having microcephaly.

Previous 2q13 CNV literature has described congenital heart defects, hypotonia and seizures as associated medical phenotypes. Combined analysis found that 31% of deletion carriers had heart defects, and this phenotype was not observed in duplication carriers. Combined analysis identified 44% of deletion carriers and 43% of duplication carriers as having hypotonia, supporting previous results on the association of the 2q13 deletion with this feature and extending this to also affect duplication carriers. Seizures were only observed in deletion carriers at a frequency of 26%. Deep phenotyping in this study also associated novel medical phenotypes with 2q13 CNVs, including: glue ear, sleep disturbances and recurrent infections of the middle ear, both in deletion and duplication carriers, and arthralgia, joint hypermobility, and gastroesophageal reflux in deletion carriers only.

A limitation of published 2q13 case reports is that many are in young children, who are below the typical assessment age for various psychiatric disorders. Also, it is unclear in some studies whether comprehensive behavioral and mental health assessments have taken place. The new cases presented in this study had a median age of 9 years, and 64% already had a clinical psychiatric diagnosis. Some of the remaining participants either had suspected psychiatric disorders, which had yet to be formally tested, or met PAS‐ADD thresholds, indicating that this figure could be even higher. To our knowledge, challenging behaviors have never previously been assessed in 2q13 CNV carriers and we found both aggressive and self‐injurious behaviors to be present in deletion carriers and self‐injurious behaviors in one duplication carrier.

Despite the aforementioned limitations, previous case reports of individuals with 2q13 CNVs have reported both ASD and ADHD diagnoses. Combining analysis identified 48% of deletion carriers and 60% of duplication carriers as having an ADHD diagnosis, and 33% of deletion carriers and 17% of duplication carriers as having an ASD diagnosis. Both 2q13 deletions and duplications have also been identified in schizophrenia patients. We did not identify any participants with schizophrenia, although only three individuals were over the age of 16, so the typical age of onset was not reached in most individuals. Our study identifies a strikingly high incidence of ADHD in 2q13 CNV carriers. A literature review of genes in the 2q13 region was undertaken and no prior association of genes in this region with risk for ADHD was identified, although postulations have been made about the involvement of genes in the region in other neuropsychiatric phenotypes.

We identified a 1.3 Mb common region of overlap in the new cases described in this study, encompassing four genes: *ACOXL*, *BCL2L11*, *ANAPC1*, and *MERTK*. It has been suggested that disruption of the *ACOXL* and *BCL2L11* genes may contribute to neurodevelopmental and ASD phenotypes (Yu et al., 2012). The *ACOXL* gene encodes a protein responsible for fatty acid oxidization, and alterations in fatty acid metabolism have been proposed to play a role in the pathogenesis of ASD (Wiest, German, Harvey, Watkins, & Hertz‐Picciotto, 2009). *BCL2L11* encodes a neuronal apoptosis regulator and previous research has found decreased expression of this gene in the frontal cortex and cerebellum of autistic subjects. It has been hypothesized that an increase in apoptosis in these regions may contribute to the pathogenicity of ASD (Sheikh et al., 2010). *ANAPC1*, a neurodevelopmental facilitator, and *MERTK*, a TAM receptor and multiple sclerosis risk gene, have also been proposed as candidate genes for the psychosis phenotype of 2q13 CNV carriers (Costain et al., 2013). All but one participant had CNVs which extend distally to include *FBLN7* and *TMEM87B*. Russell et al. (2014) undertook a functional analysis of candidate genes in the 2q13 region using zebrafish morpholino knockdowns. They found that depletion of *FBLN7* and *TMEM87B* orthologues resulted in cardiac hypoplasia and *FBLN7* depletion was also associated with craniofacial abnormalities (Russell et al., 2014).

One theory as to why some CNVs show incomplete penetrance is that a second genetic hit is required to unmask the predisposition to a neuropsychiatric phenotype (Girirajan et al., 2010). None of the participants in this study had another CNV that had been classified as pathogenic. However, as sequencing data was not available for analysis, it cannot be ruled out that another genetic variant was contributing the phenotype. Yu et al. (2016) recently identified a paternally inherited variant in the *TMEM87B* gene, one of the genes in the 2q13 region associated with the cardiac phenotype (Russell et al., 2014), in a patient with a severe cardiac phenotype who also had a maternal 2q13 deletion. It is thought that the unmasking of this homozygous variant by the maternal deletion acted as a second genetic hit, resulting in the severe phenotype (Yu et al., 2016). The inheritance pattern of 2q13 CNVs was mixed, as was the family history of ID and mental health problems. It is of interest that 25% of the new cases who had inherited CNVs had a family history of ID and mental health problems on the other side of the family from which the variant was transmitted. This could provide support to the second‐hit hypothesis, but further genetic investigations would be required.

One of the benefits of receiving a genetic diagnosis for patients and their families is the ability to access diagnosis specific information, which could be used to facilitate early intervention screening for associated medical and psychiatric phenotypes. Disorder guides, written for both professionals and families, are available for 2q13 CNVs from the patient support group unique (http://www.rarechromo.co.uk/html/DisorderGuides.asp). The findings from combined analyses in this study could also guide clinical management of individuals with newly diagnosed 2q13 CNVs. However, it must be acknowledged that while we find some phenotypes to occur more frequently, 2q13 CNV carriers still display variable phenotypic outcomes—posing challenges for genetic counseling of patients and their families. Many studies of rare CNVs have been undertaken in pediatric cohorts, and comprehensive psychiatric and behavioral phenotyping has not been carried out. The degree to which neuropsychiatric phenotypes are common in rare CNV carriers has, therefore, yet to be established. More research is required in this area and ongoing mental health assessments in 2q13 carriers will be required to elucidate associations with psychiatric disorders across the life course. Additionally, further studies of the unaffected parents and healthy controls with 2q13 CNVs will be important to elucidate potential protective factors.

The limitations of this study are that observations are being made in participants who have presented to clinical services. This may create an ascertainment bias, whereby the most severe cases are described. However, the accumulation of cases from a wide range of sources attempted to ensure as representative a sample as possible. The assessments of dysmorphology were not conducted by a clinical dysmorphology expert, although we utilized a second rater to improve the reliability of the observations. The PAS‐ADD and ChA‐PAS schedules were completed by a researcher, and clinical verification by a trained psychiatrist did not take place. Some of the participants were as young as four, meaning some of the later‐onset phenotypes could not be accurately measured at this age. However, if anything this would have led to an under estimation of the phenotype frequencies.

## 5 CONCLUSIONS

In the largest study of 2q13 CNVs to date, we present detailed phenotypic data for 25 new 2q13 deletion and duplication carriers. Combining this with previous literature yields a total of 54 deletion and 23 duplication carriers, enabling a refined understanding of the phenotypic associations of CNVs at the 2q13 locus. Combined analysis predominantly supports existing literature on an increased rate of developmental, medical, and dysmorphic phenotypes. Psychiatric investigations reveal that the majority of deletion and duplication carriers have been clinically diagnosed with a psychiatric disorder, with a particularly high incidence of ADHD. This could have important implications for psychiatric screening upon clinical diagnosis of 2q13 CNVs, and further investigation of this region may have some relevance to understanding the neurobiology of ADHD.

## Supporting information

Additional Supporting Information may be found online in the supporting information tab for this article.

Supporting Information Table I

Supporting Information Table II
